# Managing gastric cancer risk in lynch syndrome: controversies and recommendations

**DOI:** 10.1007/s10689-021-00235-3

**Published:** 2021-02-21

**Authors:** C. Richard Boland, Matthew B. Yurgelun, Kathryn A. Mraz, Patrick M. Boland

**Affiliations:** 1 Department of Medicine, University of California San Diego, San Diego, CA USA; 2 Department of Medical Oncology, Dana-Farber Cancer Institute, Boston, MA USA; 3 Center for Genomic Interpretation, Sandy, UT USA; 4 Department of Medical Oncology, Rutgers Cancer Institute of New Jersey, New Brunswick, NJ USA

## Introduction

Lynch syndrome is the hereditary predisposition to several cancers caused by pathogenic variants in the germline of certain DNA mismatch repair (MMR) genes: *MSH2* (and *EpCAM*), *MLH1*, *MSH6* and *PMS2* [1]. The greatest risks are for colorectal cancer and endometrial cancer, but other organs are also at increased risk for cancer at earlier than expected ages [2]. Surveillance colonoscopy every 1–3 years and timely gynecological surgery can significantly decrease cancer mortality in patients with Lynch syndrome. Managing cancer risk in other organs is more controversial, and a variety of management regimens have been suggested over time by different expert panels.

## Gastric cancer in Lynch syndrome

Gastric cancer in Lynch syndrome has been a controversial risk problem for a variety of reasons. The initial report of this disease occurred in 1913, when Warthin reported “Cancer Family G”. In the first reported generation of this family, gastric cancer and colorectal cancer each caused the deaths of two individuals. When the pathogenic germline variant in *MSH2* was found, the family was followed up over seven generations, and gastric cancer was the third most common malignancy in the family, although that cancer decreased in frequency over the course of the twentieth century [3]. Gastric cancer fell in incidence throughout the twentieth century in US and European populations, and perhaps this provides some explanation for why it may have fallen in Lynch syndrome families as well. The problem here is that the reported incidence of gastric cancer in Lynch syndrome ranges from 6 to 13% [4], but there may be changes in incidence over the last 100 years, and there are certainly different incidences in geographical locations or ethnic groups that have a higher background incidence of this disease [5]. The data are scant, but 62–79% of gastric cancers in Lynch syndrome are intestinal type, whereas 23–32% are reported to be diffuse or poorly differentiated; some registries do not have information on all of the tumors [6, 7]. The tumors occur in the cardia, body and antrum of the stomach [4, 7].

## Surveillance recommendations for gastric cancer risk in patients with Lynch syndrome

At this time, the most effective way to surveille patients for upper gastrointestinal cancer is through esophago-gastro-duodenoscopy (EGD). This approach permits early diagnosis in asymptomatic patients, but unlike the colon, there are no premalignant lesions to remove and reduce cancer incidence. The questions are at what age one should begin surveillance, how frequently to repeat it, and whether it is possible to identify specific subgroups of patients with Lynch syndrome for whom a different regimen is appropriate. In light of the variable estimates of the risk of gastric cancer in this setting, one challenge is to determine what threshold of cancer risk is required to trigger an invasive surveillance program. Most expert panels have suggested that endoscopic surveillance is appropriate, but recommendations differ concerning the age to initiate surveillance and the frequency of repeating the exams; moreover, some groups advise against surveillance programs (Table 1) [8–16].

**Table 1:** Surveillance recommendations for gastric cancer prevention in Lynch syndrome

Organization	Age to consider beginning EGD	Surveillance Interval	Helicobacter pylori testing?	Other
ACG [10]	30–35 years	3–5 years (see recommendation under Other)	Yes, and treat positives	Surveillance if positive family history of GC or duodenal cancer
ASCO [11]	Not stated	1–3 years in high risk populations	Yes, and treat positives	Endorsed ESMO guidelines
EHTG [8]	Not recommended	Not recommended	Yes	Consider surveillance in countries with increased GC (Korea, Japan)
ESDO [12]	30 years	1–2 years in all patients	Yes	
ESGE [16]	Not recommended	Not recommended	Yes	
ESMO [12]	Not stated	1–3 years (see recommendation under Other)	Yes	Surveillance in “high-risk populations”
NCCN [14]	40 years	3–5 years in all patients	Yes, with each EGD	Asian patients may benefit from surveillance
USMSTF [15]	30–35 years	2–3 years	Yes (biopsy)	Surveillance based upon “patient risk factors”

Adapted from Kim et al. [9]

*ACG* American College of Gastroenterology, *ASCO* American Society for Clinical Oncology, *EHTG* European Hereditary Tumour Group (formerly the Mallorca Group), *ESDO* European Society of Digestive Oncology, *ESGE* European Society of Gastrointestinal Endoscopy, *ESMO* European Society for Medical Oncology, *NCCN* National Comprehensive Cancer Network, *USMSTF* United States Multisociety Task Force, *UGI* upper gastrointestinal, *GC* gastric cancer

## Recent research on the subject of EGDs in patients with Lynch syndrome

To help illuminate this problem, Ladigan-Badura et al. evaluated the effectiveness of EGD surveillance in patients with Lynch syndrome using data from the German Consortium for Familial Intestinal Cancer that dates back to 1999 [4]. In this prospective (but non-randomized) multi-cohort study of 2009 registered people with Lynch syndrome, 1128 underwent 5176 upper endoscopic exams at the time of their surveillance colonoscopies, which were typically done every 1–3 years. The investigators observed 49 gastric cancers in 47 patients. Patients undergoing surveillance EGDs were significantly more likely to be diagnosed with early stage disease (IUCC 1a-1b) than in those diagnosed because of symptoms (83% vs 25%, P = 0.23). Most of the patients (68%) reported no family history of gastric cancer. The median age for the diagnosis of gastric cancer was 51 years (range 28–66), and 13 (28%) were younger than age 45. Almost all cases were in patients with pathogenic variants in *MSH2* or *MLH1*; one was related to *MSH6*, one with *EpCAM*, and none were reported in *PMS2* patients. Males made up 62% of the gastric cancer group. The authors conclude that finding significantly fewer advanced gastric cancers demonstrates the effectiveness of screening EGDs, and recommended routine surveillance in Lynch syndrome beginning at age 30.

## The role of *H. pylori* and gastritis

An issue not addressed here is the value of testing for *H. pylori* infection at the time of the EGD. Although this does not add serious time or morbidity to an EGD and is listed in virtually all of the guidelines (Table 1), there is not much evidence that this is actually helpful. A recent study by Kumar et al. reported that in their cohort of 295 patients with Lynch syndrome who underwent 660 EGDs, 6 gastric cancers were found (2.8%), and just 6 in the whole cohort (again 2.8%) were *H. pylori* carriers. Although not specifically stated in the publication, none of the gastric cancers occurred in the context of a *H. pylori* infection (personal communication, BW Katona) [17]. This group also reported that 4 of the 5 upper gastrointestinal cancers detected on surveillance occurred within 2 years of the prior EGD [17]. Perhaps surveillance intervals require additional scrutiny. Equivalent rates of *H pylori* infection have been reported for patients with Lynch syndrome with or without a first degree relative gastric cancer [18]. Also, it has been recently reported in a small series that gastric cancer in Lynch syndrome is associated with underlying chronic autoimmune gastritis unrelated to *H. pylori* infection, which opens another avenue for screening and risk assessment [19].

## Proposed conclusions

What can the reader conclude from the study by Ladigan-Badura and colleagues? First, it takes a lot of EGDs to find early stage asymptomatic gastric cancers in Lynch syndrome. A diagnosis of gastric cancer was made in only 47/2009 (2.3%) of all their patients and in 48/5176 (0.93%) of their exams. That is a lot of negative exams. However, given the lethality of an advanced gastric cancer, this may be acceptable, especially since the exam could be done on an already sedated patient who is in the endoscopy suite for a colonoscopy, which should add only a few minutes and minimal morbidity to the effort. Secondly, the findings here and elsewhere [6] indicate that only a minority of individuals with Lynch syndrome-associated gastric cancer will have a family history of gastric cancer. Although a recent study found that a patient with Lynch syndrome is significantly and incrementally more likely to develop gastric cancer when there are first degree relatives with gastric cancer, more than two thirds of patients with Lynch syndrome-associated gastric cancers have no family history, so a reliance on this finding alone to guide EGD surveillance would likely miss the majority of cases [9]. Additionally, there would appear to be reason to focus this surveillance on patients with Lynch syndrome who carry pathogenic variants in *MSH2*, *MLH1* and (probably) *EpCAM*, but not do this on patients with *MSH6* pathogenic variants (probably) and *PMS2* (certainly). Using large genetic testing panels will also lead to the identification of lower penetrance genes and pathological variants in genes that are not suspected to raise the risk of gastric cancer. Future research is required to determine the optimal response to these situations [20].

## Final caveat

Finally, this work stems from a European population, and one cannot assume that the data would be similar in countries with very high risks for gastric cancer, such as Japan and Korea, where the clinical picture appears to be quite different [21].
